# Women, tobacco, and human rights

**DOI:** 10.18332/tid/137473

**Published:** 2021-06-10

**Authors:** Kelsey Romeo-Stuppy, Laurent Huber, Patricia Lambert, Mira B. Aghi, Soon-Young Yoon, Daniel Thomas, Gabrielle Ballweg

**Affiliations:** 1 Action on Smoking and Health, Washington, United States; 2 International Legal Consortium, Campaign for Tobacco-Free Kids, Washington, United States; 3 Healis Sekhsaria Institute of Public Health, Navi Mumbai, India; 4 Human Rights Tobacco Control Network, Washington, United States; 5 International Alliance of Women, New York, United States; 6 Women’s Environment and Development Organization, New York, United States; 7 Institute of Cardiology, Hôpital Pitié-Salpêtrière, Sorbonne University, Paris, France; 8 Alliance Against Tobacco, Paris, France

**Keywords:** women’s rights, intersectoral partnerships, tobacco, WHO FCTC, CEDAW

The tobacco industry violates the human rights of women and girls. To combat the industry’s massive women’s rights violations, input from civil society, such as the International Network of Women Against Tobacco (INWAT), has placed women at the heart of the WHO Framework Convention on Tobacco Control (FCTC). By highlighting essential female participation in policymaking and gender-specific tobacco control policies, the WHO FCTC contests tobacco consumption among women and girls. Article 4.2(d) of the WHO FCTC includes a direct reference to gender-specific risks when developing tobacco control strategies, and Article 4.7 encourages civil society involvement in WHO FCTC implementation, including women’s organizations. Furthermore, the Preamble makes specific reference to the need to harmonize the WHO FCTC with other human rights treaties, notably the Convention on the Elimination of All Forms of Discrimination against Women, the Convention on the Rights of the Child, and the Covenant on Economic, Social and Cultural Rights^1^.

The prevalence of tobacco use is declining due to the WHO FCTC and other collective actions against the tobacco industry. However, this decline is unequal: as of 2021, tobacco use among women is decreasing at a significantly lower rate than in men, and in some countries, women are smoking more. In France between 1980 and 2012, despite a 6.3% decrease in smoking among men, there was a 75% increase in smoking among women^2^. Furthermore, the percent of female deaths attributable to tobacco was nine times greater in 2010 in comparison to 1980 levels^3^. Since 2000, the number of tobacco-related deaths in women aged under 65 years who actively use tobacco products has more than doubled^4^. Other health consequences in women due to tobacco use including dramatic increases in heart attacks^5^, strokes^6^, lung cancer, and COPD, have exacerbated morbidity^4^. Since 2016, the smoking prevalence in France has decreased, but 20.7% of women in France are still daily smokers^7^. Globally, women are still the main victims of secondhand smoke and more women than men are harmed or die from secondhand smoke^8^.

However, active smoking is not the only concern when it comes to women’s rights and tobacco. In India, bidi cigarettes account for 81% of tobacco smoking^9^, and 90% of bidi producers are women^10^. Labor unions and nongovernmental organizations (NGOs) estimate that nearly 10 million Indian women and children are rolling bidis annually. These child workers are generally girls and have no wage structure, often earning less than other bidi producers who make just over two dollars in a 12-hour workday^10^. Girls often drop out of school and learn bidi rolling to help their mothers who cannot keep up with the work^11^. Since these girls enter bidi rolling as children, they have little education and few alternatives than to becoming bidi rollers themselves. Besides its impact on women’s education, the inhalation of bidi dust and working in a crouched position is detrimental to their health^11^. During the COVID-19 pandemic, bidi rolling became the primary income source for some families when men lost their jobs^12^.

This pandemic and the global climate emergency have accentuated underlying inequalities: women’s disproportionate burden of work, their minimal social protection, and gender-based violence. For this reason, the International Alliance of Women (IAW), an NGO dedicated to promoting the human rights of women and girls, recognizes the need to integrate strategies on gender, women, and tobacco control within the context of the broader sustainable development goals related to poverty reduction, employment, and environment. Women’s rights movements should incorporate tobacco control to build constructive dialogue and find a nexus in action points between women’s human rights and United Nations (UN) events. Civil society must work at the UN to accelerate efforts to combat these large-scale simultaneous crises from the perspective of women and tobacco control.

The Generation Equality Forums (GEFs) for Beijing+25 convened by Mexico and France in 2021 are opportunities for civil society to integrate women’s rights and tobacco control. The Forums are civil society driven, co-sponsored by Mexico and France, and convened by UN women. They aim to bridge the gap between multiple generations and promote innovative partnerships between member states, the private sector and philanthropies, international organizations including the UN, and civil society. The events summarize progress towards the implementation of the Beijing Platform for Action, CEDAW, and the 2030 Agenda outside of the intergovernmental space while accelerating action on the UN agenda. Tobacco control is directly related to three of the six action coalitions that provide the foundation for these Forums: reproductive health and rights, economic justice, and gender-based violence^13^.

Another opportunity to engage with the feminist and women’s movements beyond the Commission on the Status of Women, the GEFs, and regional meetings, includes the High-Level Political Forum, which monitors the UN 2030 Agenda for Sustainable Development^14^. By joining any of the nine major groups, NGOs have opportunities to present a collective voice to member states during the Forum. The inclusion of tobacco control policies in these groups is instrumental to achieving the Sustainable Development Goals on health, gender equality, sustainable cities, and climate action, among many others^15^.

Tobacco control advocates can also combat tobacco as a women’s rights issue on the international and local level through the International Convention on the Elimination of All Forms of Discrimination against Women (CEDAW). The Convention is ratified by 189 countries and is similar to an international bill of rights for women. CEDAW’s Article 10(h) discusses access to specific educational information, as education on tobacco is necessary when dealing with the industry. Article 11(f) emphasizes the right to protection of health and safe working conditions. An unsafe work environment contributes to women accounting for 64% of all deaths due to passive smoking^8^.

On an international level, advocates can urge the committee to act on women’s rights and tobacco by submitting a shadow report to CEDAW that recommends considering tobacco control when implementing human rights treaty bodies^16^. After submission, advocates can also make an oral statement to the committee. By using human rights language within CEDAW, tobacco control organizations can persuade local governments to act on tobacco’s massive women’s rights violations. Furthermore, fostering intersectoral connections, especially among women’s rights groups, elevates both tobacco control and women’s rights on the local and international level.

There is an opportunity for women in tobacco control. Collective action against the tobacco industry is necessary to support gender-specific tobacco control policies. Together, women’s groups and tobacco control advocates can foster the political will necessary to end the tobacco epidemic and its disproportionate impact on women and girls.
